# Molecular characterization, comparative genome analysis and resistance determinants of three clinical *Elizabethkingia miricola* strains isolated from Michigan

**DOI:** 10.3389/fmicb.2025.1582121

**Published:** 2025-07-09

**Authors:** Shicheng Chen, Grace Agah, Jochen Blom, Edward D. Walker

**Affiliations:** ^1^College of Health and Human Sciences, Northern Illinois University, DeKalb, IL, United States; ^2^Bioinformatics and Systems Biology, Justus-Liebig University Giessen, Giessen, Germany; ^3^Department of Microbiology, Genetics, and Immunology, Michigan State University, East Lansing, MI, United States

**Keywords:** *Elizabethkingia miricola*, genome analysis, antimicrobial resistance, molecular identification, pangenomes

## Abstract

**Introduction:**

*Elizabethkingia miricola* is a gram-negative bacterium that causes life-threatening infections in vulnerable populations. Unlike other species in the *Elizabethkingia* genus, *E. miricola* also leads to meningitis-like diseases in aquatic invertebrates such as frogs, raising concerns about its zoonotic transmission potential. Management of its infection is complicated by unclear transmission pathways and multi-drug resistance.

**Methods:**

In this study, we analyzed three clinical strains (*E. miricola* Mich-1, Mich-2, and Mich-3) isolated from patients in Michigan using morphology observations, biochemical tests, matrix-assisted laser desorption/ionization time-of-flight mass spectrometry (MALDI-ToF/MS), and genome sequencing.

**Results:**

Average Nucleotide Identity (ANI) analysis revealed that the Michigan strains were nearly identical and shared 96.52% identity with the type strain *E. miricola* DSM 14571, confirming their classification as *E. miricola*. Comprehensive comparative genomic analyses were conducted across 28 strains, including human isolates and strains from invertebrates like frogs. The strains exhibited open pan-genome characteristics. Mich-1 shared 3,199 genes (83.2%) with human isolates but fewer genes with frog-derived isolates (ranging from 3,319 to 3,375). This phylogenetic analysis highlights regional variation and the global diversity of *E. miricola* isolates, revealing connections between clinical and environmental strains. Antibiotic susceptibility testing revealed that the three clinical strains were resistant to 13 out of 16 tested drugs, with susceptibility only to trimethoprim/sulfamethoxazole and ciprofloxacin. The strains carried five β-lactamase-encoding genes (*BlaB-10*, *BlaB-39*, *CME-1*, *CME-2*, and *GOB-25*), conferring resistance to penams, cephalosporins, and carbapenems. Several virulence-associated genes were conserved across clinical and frog isolates. These genes contribute to stress adaptation, adherence, and immune modulation.

**Discussion:**

This study underscores the evolutionary adaptability of *E. miricola* genomes, highlighting their capacity to acquire genetic traits that enable survival in diverse niches. This adaptability facilitates the emergence of more resistant and virulent strains, posing significant threats to both human and animal health.

## 1 Introduction

*Elizabethkingia* is a genus of gram-negative, non-fermenting, aerobic rod (Breurec et al., 2016; Coyle, 2017; Janda and Lopez, 2017). Bacteria *Elizabethkingia* are widely distributed in various environments such as soil, water, plants, and animals (Lin et al., 2019). At least seven species have been identified with medical importance, including *E. anophelis*, *E. miricola, E. meningoseptica, E. occulta, E. bruuniana, E. ursingii*, and *E. umeracha* (Nicholson et al., 2018; Hem et al., 2022). Among them, *E. anophelis* is more frequently isolated from human specimens, followed by *E. miricola* and *E. meningoseptica* (Coyle, 2017; Janda and Lopez, 2017; Lin et al., 2019; Hem et al., 2022; Zajmi et al., 2022). Different from other *Elizabethkingia*, *E. miricola* causes serious acute infections in both humans and aquatic invertebrate animals (Ransangan et al., 2013; Zajmi et al., 2022; Wu et al., 2024). The common clinical presentations in humans include bacteremia, pneumonia, sepsis, and meningitis (Coyle, 2017; Janda and Lopez, 2017; Lin et al., 2019; Lee and Hsueh, 2023; Wu et al., 2024). Overall, the elderly, neonates, immunosuppressed patients, and individuals with underlying chronic medical conditions are more susceptible to *E. miricola* infections (Lin et al., 2019; Huang et al., 2024). In anuran species, it caused meningitis-like diseases in bull frogs, northern leopard frogs (*Lithobates pipiens*), Chapa bug-eyed frogs (*Theloderma bicolor*), and Vietnamese warty toads (*Bombina microdeladigitora*) (Zajmi et al., 2022). Additionally, *E. miricola* was also isolated from Tra catfish (*Pangasius hypophthalmus*) filets in the industrial processing lines in Vietnam (Ransangan et al., 2013; Zajmi et al., 2022).

Managing *E. miricola* infections has been particularly challenging due to its multidrug resistance (MDR) (Comba et al., 2022; Wu et al., 2024). This bacterium exhibits intrinsic resistance to a wide range of important antibiotics that are used for treating infections by gram-negative bacteria (Wu et al., 2024). The MDR mechanisms remain unexplored in *E. miricola.* However, many reports showed that the MDR in *E. anophelis* is primarily mediated by chromosomally encoded determinants (Comba et al., 2022; Wu et al., 2024). Its resistance extends to nearly all β-lactam antibiotics, driven by the presence of three distinct β-lactamase genes: *blaCME*, an Ambler class A serine extended-spectrum β-lactamase (ESBL), and *blaB* and *blaGOB*, which encode Ambler class B metallo-β-lactamases (MBLs) (Hu et al., 2020; Yang et al., 2021; Comba et al., 2022; Andriyanov et al., 2024). The dissemination of resistance genes is largely facilitated by mobile genetic elements such as conjugative transposons and prophages, which carry genes encoding efflux pumps, enzyme-degrading proteins, and enzyme-modifying proteins, further complicating treatment options (Comba et al., 2022; Andriyanov et al., 2024; Huang et al., 2024). Large-scale outbreaks and the global distribution of this group of pathogens have been observed (Breurec et al., 2016; Perrin et al., 2017; McTaggart et al., 2019; Hu et al., 2022b; Mallinckrodt et al., 2023; Wu et al., 2024). The resistance to antimicrobials and disinfectant treatment phenotypes have been linked with the formation of biofilms (Hu et al., 2022a; Puah et al., 2022). Compounding these challenges, *E. miricola* is often misclassified as *E. meningoseptica* or *E. anophelis*, indicating that the infections by *E. miricola* have been underestimated (Andriyanov et al., 2024; Huang et al., 2024; Wu et al., 2024). Antibiograms of *Elizabethkingia* isolates often reveal inconsistent resistance patterns, underscoring the need for local susceptibility testing to guide effective treatment (Huang et al., 2024). Infections caused by *Elizabethkingia* spp. are associated with increased mortality when inappropriate antimicrobial therapy is administered (Huang et al., 2024). Accurate identification of this clinically significant pathogen in time and understanding its MDR mechanisms are particularly important for improving the management of *Elizabethkingia* infections (Coyle, 2017; Janda and Lopez, 2017; Huang et al., 2024).

Three *E. miricola* strains were detected in Michigan patients during the same period as the large *Elizabethkingia* outbreak that occurred in the United States between 2015 and 2016 (Perrin et al., 2017). During that time, most attention was drawn to *E. anophelis* (Perrin et al., 2017). Outbreaks of *E. miricola* infections in humans have been poorly documented, with only a small outbreak reported in intensive care units in Spain in 2021 (Soler-Iborte et al., 2024). So far, there were at least 9 available genomes of *E. miricola* deposited in the GenBank with most isolated from Midwest regions in United States. The transmission pathways of *E. miricola* remain unclear, though current research suggests several potential routes (Hu et al., 2020; Zajmi et al., 2022; Huang et al., 2024; Wu et al., 2024). Healthcare-associated transmission is considered the most plausible, supported by its detection in hospital settings and its similarity to *E. anophelis* and *E. meningoseptica*, which have been linked to outbreaks via contaminated medical devices and inadequate sterilization practices (Coyle, 2017; Janda and Lopez, 2017; Lin et al., 2019; Hu et al., 2022a). However, many affected patients have neither been hospitalized nor lived in long-term healthcare facilities, indicating alternative routes (Coyle, 2017; Janda and Lopez, 2017; Lin et al., 2019; Mallinckrodt et al., 2023). Environmental exposure is another possibility, as *E. miricola* has been isolated from freshwater, soil, animals, and plants, highlighting its resilience in both natural and built environments (Lin et al., 2019; Kadi et al., 2022; Zajmi et al., 2022). Zoonotic transmission is also suggested by its presence in diseased animals, such as cultured fish and frogs, pointing to potential environmental or direct animal-to-human transmission (Lin et al., 2019; Puah et al., 2022; Zajmi et al., 2022). While direct evidence of person-to-person transmission is lacking, outbreaks of related species in healthcare settings suggest that such transmission, possibly involving healthcare workers, could occur, especially among immunocompromised individuals (Lin et al., 2019; Puah et al., 2022; Zajmi et al., 2022).

Epidemiological studies, genomic analyses, and expanded environmental sampling are essential to better understand the transmission pathways, MDR mechanisms, and strategies for disease management of *E. miricola* (Hem et al., 2022; Huang et al., 2024). In this study, we sequenced three *E. miricola* strains collected during a cluster outbreak in Michigan. A detailed phylogenetic analysis was conducted to compare genetic variations between strains isolated from amphibians and those from clinical settings. Furthermore, we investigated the molecular mechanisms underlying drug resistance in this emerging pathogen. Our research on the geographical distribution, phylogenetic structure, and MDR mechanisms of *E. miricola* provides valuable insights into its drug resistance and virulence factors, as well as predictions regarding host-pathogen interactions and host-environment responses.

## 2 Materials and methods

### 2.1 Culture

Three strains of *E. miricola* (Mich-1, Mich-2, and Mich-3) were isolated from patients in Michigan (see Table 1). Strain Mich-1 was obtained from the whole blood of a female patient on February 22, 2016, while strains Mich-2 and Mich-3 were isolated from whole blood samples of different male patients on November 10, 2015. The *E. miricola* strains were cultured aerobically in tryptic soy broth (TSB) at 30°C. When using tryptic soy agar (TSA), Bacto agar (Difco, Detroit, MI) was added to TSB at a final concentration of 20 g/liter. Sheep blood agar (SBA) was from Thermo Scientific (Waltham, MA). *Streptococcus pneumoniae*, *Streptococcus pyogenes*, and *Enterococcus faecalis* were employed as controls for alpha, beta, and gamma hemolysis, respectively. Hemolysis patterns were identified after the tested strains were cultured on blood agar plates at 37°C for 48 h. To characterize the biochemical properties of Mich-1, we inoculated 150 μL of the bacterial suspension into a Biolog GEN III microplate (Biolog Inc., Hayward CA) and incubated it at 30°C. Color changes in plate wells were analyzed according to the manufacturer’s instructions.

**Table 1:** Genomic features in selected *Elizabethkingia* species.

List of genomes	Origin	Isolation source	Clinical specimen	Isolation date	Genome size (Mb)	GC content (%)	Total genes	CDS	CRISPR/Cas systems
EM_CHUV	Switzerland	*Homo sapiens*	Endotracheal secretions	2014	4.29	36.0	3,896	3,841	0
CIP111047	France	*Homo sapiens*	Blood	1982	4.45	35.9	4,179	4,124	1
G4071	France	*Homo sapiens*	Tracheal exudate	1978	4.27	35.9	3,924	3,874	1
G4074	UK	*Homo sapiens*	NA	NA	4.27	35.9	3,911	3,862	2
NCTC11305	UK	*Homo sapiens*	Tracheal exudate	1978	4.26	35.9	4,097	4,023	1
G4121	Sweden	*Homo sapiens*	NA	1982	4.42	35.9	4,090	4,040	1
EM 15	Brazil	*Homo sapiens*	Tracheal secretion	2016	4.48	35.8	4,107	4,055	1
CSID_3000516998	USA: SC	*Homo sapiens*	NA	2016	4.37	36	4,068	4,017	0
CSID_3000517120	USA: MN	*Homo sapiens*	NA	2016	4.43	35.9	4,035	3,984	1
SBRL-21-086	USA: OH	*Homo sapiens*	Sputum	2021	4.31	35.7	4,024	3,971	2
SBRL-21-012	USA: OH	*Homo sapiens*	Sputum	2022	4.14	35.7	3,847	3,800	0
SBRL-21-030	USA: OH	*Homo sapiens*	Wound	2022	4.09	35.8	3,721	3,675	2
CSID_3000516464	USA: MI	*Homo sapiens*	NA	2016	4.19	35.8	3,840	3,796	0
Mich-1	USA: MI	*Homo sapiens*	Blood	2016	4.19	35.8	3,844	3,799	0
Mich-2	USA: MI	*Homo sapiens*	Blood	2015	4.19	35.8	3,846	3,799	0
Mich-3	USA: MI	*Homo sapiens*	Blood	2015	4.19	35.8	3,845	3,802	0
LDVH 337.01	France	Frog	Xenopus laevi: spleen		4.15	35.9	3,746	3,687	0
IMT47318	Germany: Berlin	Frog	*Lithobates pipiens*	2019	4.29	35.8	3,879	3,777	0
IMT47357	Germany: Berlin	Frog	*Pipa parva*: heart	2019	4.29	35.8	3,851	3,775	0
IMT47538	Germany	Frog	*Lithobates pipiens*	2019	4.24	35.9	3,779	3,689	0
MEYL_1	Japan	Frog	Aquarana catesbeiana	2021	4.24	35.8	3,901	3,836	0
Mir-N11	China	Frog	NA	2021	4.31	35.7	3,973	3,901	0
NW-2-4	China	Frog		2021	4.24	35.8	3,930	3,864	0
F13	China	Frog	NA	2017	3.74	36.7	3,761	3,757	0
FL160902	China	Frog	NA	2016	4.22	35.7	3,847	3,797	0
QZY. EM	China	Frog	*Pelophylax nigromaculatus*: brain	2016	4.21	35.6	3,832	3,781	0
DSM 14571	Russia	Condensation water	NA	2001	4.3	35.8	3,977	3,925	0
BM10	South Korea	Termite	Reticulitermes speratus: Hindgut	2009	4.24	35.7	3,895	3,823	1

### 2.2 MALDI-ToF MS analyses

Pure strains of *E. miricola* were grown on sheep blood agar plates at 35.5°C for 24 h. A single colony was then smeared onto a metal target plate. Following this, 1 μL of α-cyano-4-hydroxycinnamic acid matrix solution was applied to the smeared area and allowed to dry. The target plate was subsequently placed into the VITEK mass spectrometer, a MALDI-TOF/MS system (BioMérieux, Durham, NC, United States). The resulting spectra, covering a mass range of 2 to 20 kDa, were analyzed and compared to the reference spectra of known species in the VITEK MS MS-ID database (version 2.0) for accurate identification.

### 2.3 Antibiotic susceptibility testing

A 0.5 McFarland bacterial suspension was prepared using a 24-h culture and transferred into an AST-GN69 card, which was then loaded into a VITEK 2 system (BioMérieux, Durham, NC, United States). The following antimicrobials were tested: piperacillin, piperacillin/tazobactam, trimethoprim/sulfamethoxazole, ampicillin, ampicillin/sulbactam, meropenem, aztreonam, Cefotaxime, ceftriaxone, cefazolin, amikacin, gentamicin, tetracycline, tigecycline, ciprofloxacin, and nitrofurantoin. The interpretation of results was based on standards recommended by the Clinical and Laboratory Standards Institute (CLSI) for non-Enterobacteriaceae.

### 2.4 Genomic DNA extraction, genome sequencing, assembly, and annotation

DNA was extracted using the Wizard Genomic DNA Purification Kit (Promega, Madison). The concentration of genomic DNA was quantified with a Nanodrop2000 UV–Vis Spectrophotometer (Thermo Scientific) and a Qubit DNA assay kit. DNA integrity was assessed via a 1.5% (w/v) agarose gel assay. Next-generation sequencing (NGS) libraries were prepared using the Illumina TruSeq Nano DNA Library Preparation Kit. The completed libraries were evaluated with Qubit dsDNA HS, Caliper LabChipGX HS DNA, and Kapa Illumina Library Quantification qPCR assays. The libraries were pooled for multiplexed sequencing and loaded onto a single standard MiSeq flow cell (v2). Sequencing was conducted in a 2 × 250 bp paired-end format using a v2 500-cycle reagent cartridge. NGS libraries were sequenced using Illumina MiSeq paired-end sequencing technology at the Research Technology Support Facility (RTSF) of Michigan State University. The sequencing reads were assembled using the CLC Genomics Workbench v. 23.05. The assembled genome sequences for Mich-1, Mich-2 and Mich-3 were submitted to the Prokaryotic Genome Automatic Annotation Pipeline (PGAAP 3.3) available in National Center for Biotechnology Information (NCBI) for annotation (Tatusova et al., 2016). The predicted CDSs were translated and analyzed against the NCBI non-redundant database, Pfam, TIGRfam, InterPro, KEGG and COG (Tatusova et al., 2016).

### 2.5 Bioinformatics

Genomes of 28 *E. miricola* strains and two other clinically important *Elizabethkingia* species, *E. anophelis* and *E. meningoseptica*, were downloaded from GenBank (NCBI) and reannotated using Prokka, a rapid prokaryotic genome annotation tool (Seemann, 2014). The multi-drug resistance genes were predicted in the CARD database (Alcock et al., 2023). Prophage and Clustered Regularly Interspaced Short Palindromic Repeats (CRISPR) were predicted using CRISPRfinder (Grissa et al., 2007). The virulence factors of *Elizabethkingia* species were predicted using the VFDB (Chen et al., 2016). For genome similarity assessment, average nucleotide identity (ANI) and digital DNA–DNA hybridization (dDDH) values were computed using the web tools ANI calculator (Yoon et al., 2017) and GGDC 3.0 (Meier-Kolthoff et al., 2022), respectively. For quantification and type of prokaryotic regulatory system proteins, web tool P2RP was used (Barakat et al., 2013). The pan-genome, core genome, and specific genes of Michigan isolates were analyzed by comparison with other representative *Elizabethkingia* using EDGAR 3.2 (Dieckmann et al., 2021). The sizes of pan-genome and core genomes were approximated using the core/pan development feature. The *Elizabethkingia* pangenome was further calculated using Roary v3.13.0 (Page et al., 2015) built in Galaxy^1^ and visualized using Phandango v1.3.1 (Hadfield et al., 2018).

### 2.6 Accession of genome sequences

Data from the whole-genome shotgun projects were deposited at DDBJ/ENA/GenBank for *E. miricola* Mich-1, Mich-2, and Mich-3 under accession numbers JBEUGN000000000, JBEUGO000000000, and JBEUGP000000000, respectively. BioProject numbers are PRJNA1125339, PRJNA1125343, and PRJNA 746122 and the BioSample accession numbers are SAMN41894042, SAMN41894043, and SAMN 20181758, respectively.

## 3 Results

### 3.1 Physiological and biochemical characteristics of *Elizabethkingia miricola* Mich-1, Mich-2, and Mich-3

**Figure 1:**
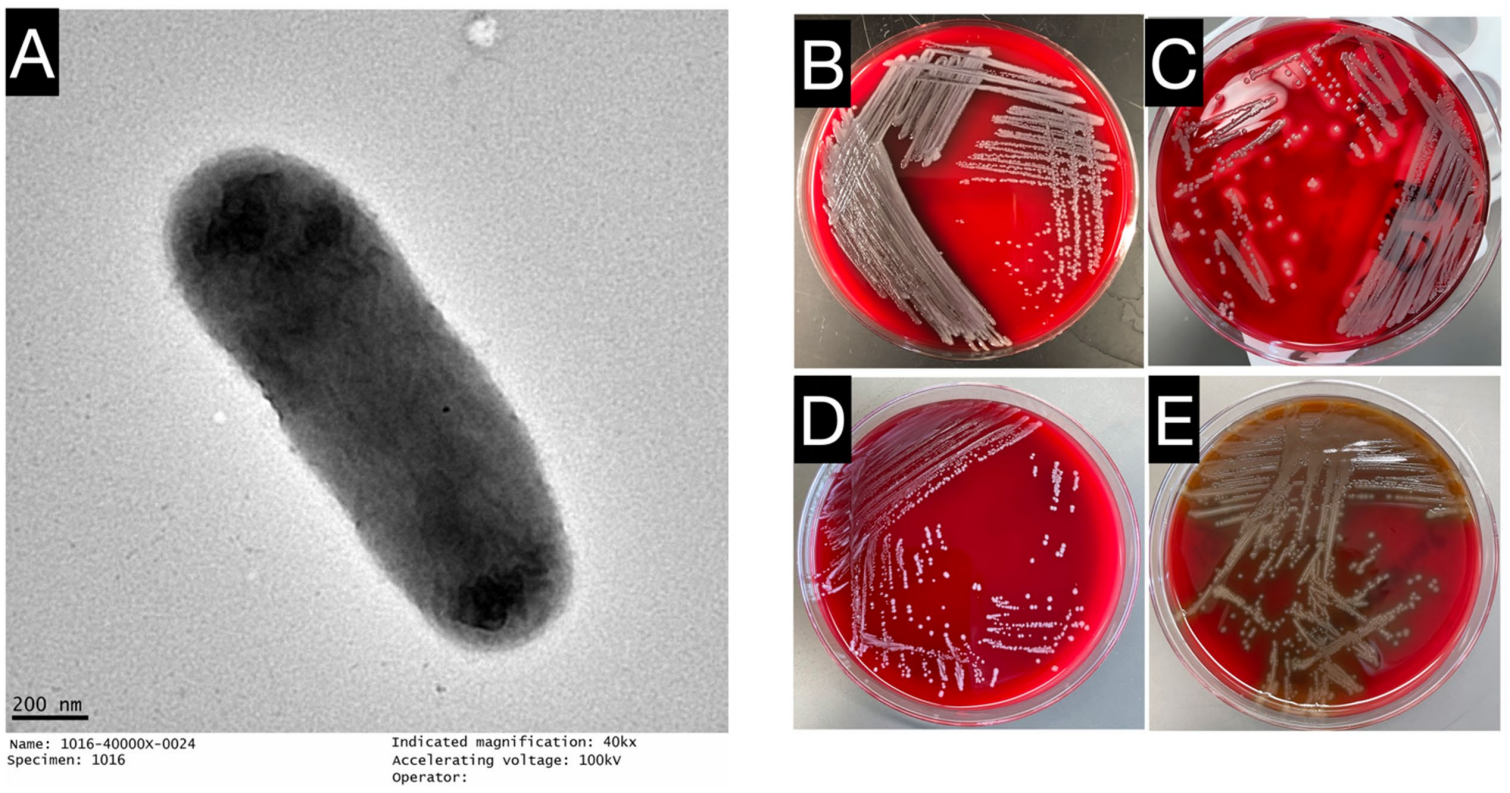
Growth characteristics and microscopic observations of *E. miricola* Mich-1. **(A)** Electron microscopy image of *E. miricola* Mich-1, visualized with a negative stain to highlight bacterial morphology. **(B)** Growth of *E. miricola* Mich-1 on sheep blood agar, demonstrating gamma hemolysis (non-hemolytic). **(C)** *S. pyogenes* on sheep blood agar, showing complete hemolysis, used as a positive control for beta-hemolysis. **(D)** *E. faecalis* on sheep blood agar, demonstrating gamma hemolysis (non-hemolytic), used as a non-hemolytic control. **(E)** *S. pneumoniae* on sheep blood agar, exhibiting alpha hemolysis (partial hemolysis), used as a control for partial hemolysis.

The three clinical isolates *E. miricola* Mich-1, Mich-2, and Mich-3 exhibited similar morphological characteristics: colonies were medium-sized, creamy, slightly mucoid, and round. Strain Mich-1 was selected as the representative for further identification. Mich-1 cells showed a straight rod with a smooth surface and defined cell borders and had a diameter of 0.4 μm and length of 15.0 μm (Figure 1A). It showed gamma hemolysis (no hemolysis) after incubation on sheep blood agar at 35°C for 24 h (see Figure 1B) while *S. pyogenesis, E. faecalis* and *S. pneumoniae* demonstrated beta, gamma and alpha hemolysis, respectively (Figures 1C–E). We assessed the ability of Mich-1 to utilize various carbon and nitrogen sources, as well as its tolerance to salt, pH, and surfactants, using Biolog GEN III microplates (Supplementary Table S1). The results demonstrated that Mich-1 metabolized a range of carbohydrates, including dextrin, d-maltose, d-trehalose, gentibiose, d-lactose, d-melibiose, d-glucose, N-acetyl-d-glucosamine, d-mannose, d-galactose, d-fucose, l-fucose, d-fructose, d-mannitol, d-arabitol, and d-glycerol. In addition, it utilized various nitrogen sources, such as gelatin, glycyl-l-proline, l-alanine, l-arginine, l-aspartic acid, l-glutamic acid, l-histidine, l-pyroglutamic acid, l-serine, pectin, d-galacturonic acid, l-galactonic acid lactone, and d-glucuronic acid. However, its growth was inhibited by 4% NaCl, the surfactant Niaproof 4, or pH levels below 5.0. These findings suggest that Mich-1 is capable of surviving in diverse environments. However, all three strains Mich-1, Mich-2 and Mich-3 were misidentified as *E. meningosepticum* by the MALDI-TOF/MS analysis (see Supplementary Figure S1).

### 3.2 Genomic features and phylogenetic inferences

**Figure 2:**
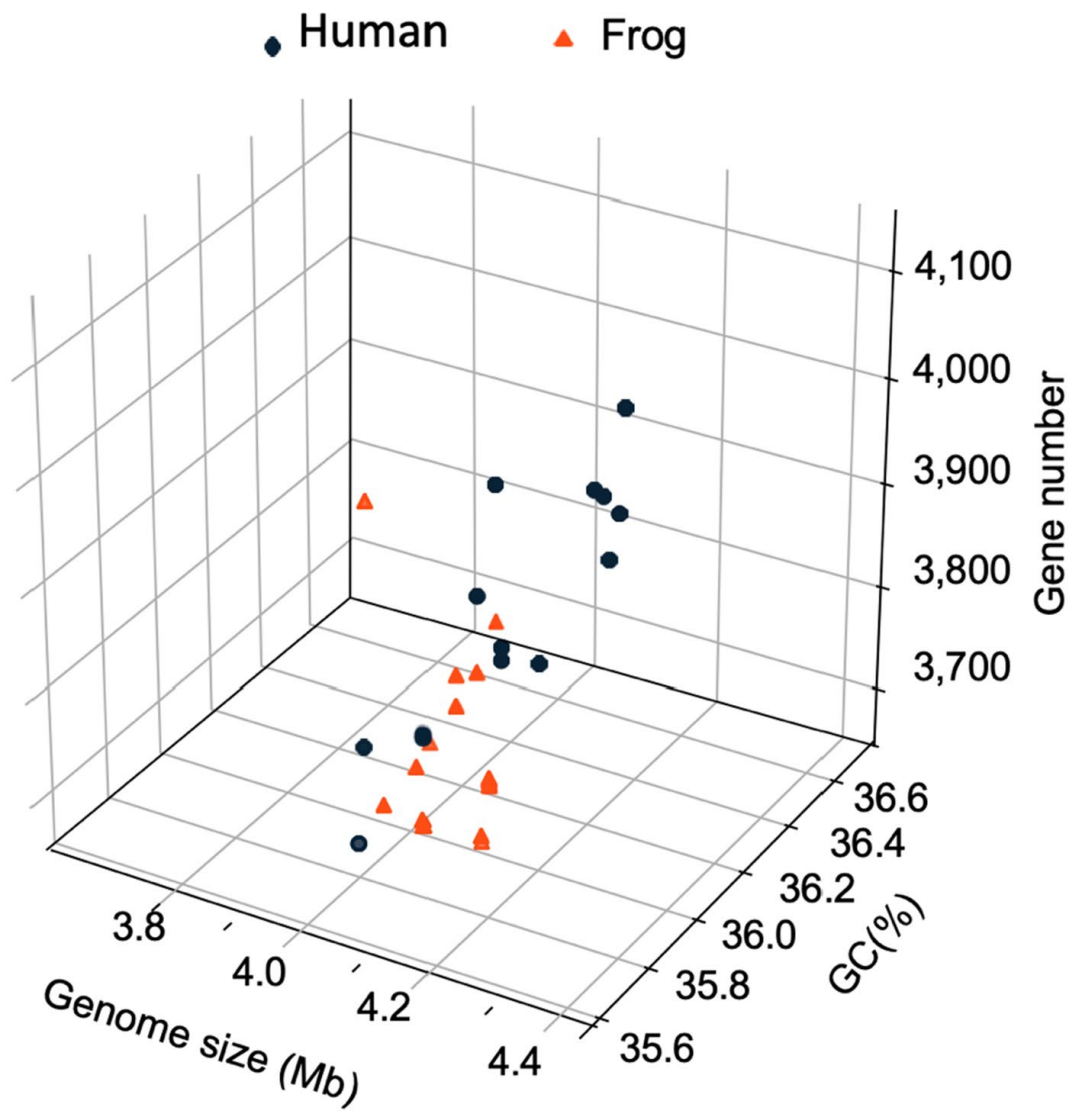
Three-dimensional plots of genome feature in selected *E. miricola* isolates. A 3D scatter plot illustrating the relationship between genome size (x-axis), coding sequence (CDS) count (y-axis), and GC content (z-axis) across the selected *E. miricola* isolates.

The genomic characteristics of the *Elizabethkingia* strains are summarized in Table 1. The assemblies of Mich-1, Mich-2, and Mich-3 contained 10, 14, and 11 contigs, respectively, with similar genome sizes of 4.19 Mb (Table 1). Among the 28 selected *E. miricola* genomes, sizes ranged from 3.74 Mb to 4.48 Mb, with an average size of 4.25 Mb. Notably, the average genome size of isolates from U. S. patients (4.23 Mb) was slightly smaller than those from European and Brazilian patients (4.34 Mb), a difference that was statistically significant (*p* < 0.05). However, no significant difference in genome size was observed between isolates from humans and frogs (*p* > 0.05). A similar trend was observed in the total predicted gene numbers across these genomes. The genomes of Mich-1, Mich-2, and Mich-3 contained 3,799, 3,799, and 3,802 coding sequences (CDSs), respectively. The average GC content of the three *E. miricola* strains isolated from Michigan patients was 35.8%, consistent with the average GC content of isolates from U. S. patients (35.81%), but slightly lower than that of European and Brazilian isolates (35.9%; p < 0.05). Additionally, the three Michigan isolates lacked predicted CRISPR elements (Table 1). Overall, the genomes of clinical isolates showed high similarity to those from frog isolates (Figure 2).

**Figure 3:**
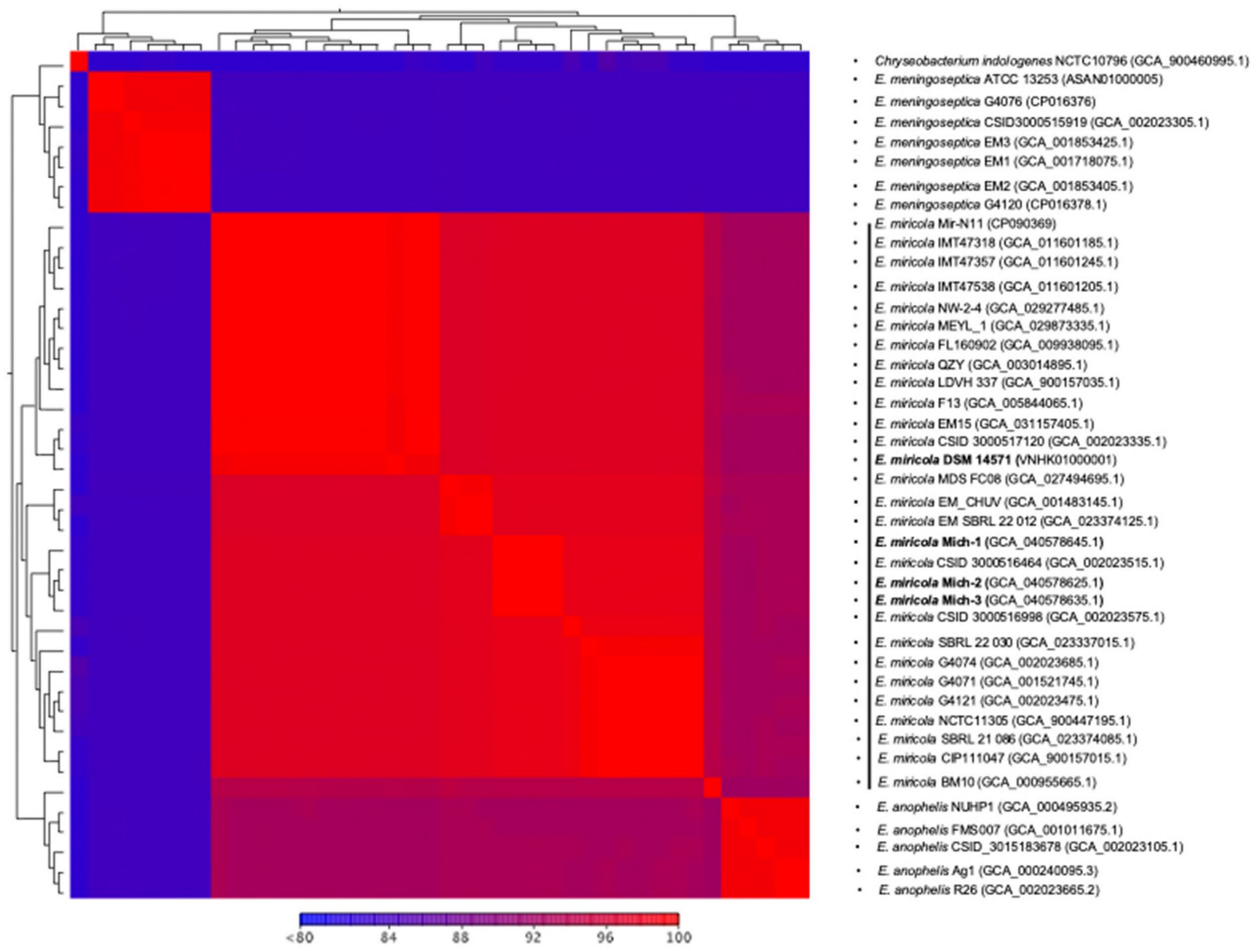
Heatmap of pairwise ANI values of selected *Elizabethkingia* genomes. Heatmap showing the pairwise Average Nucleotide Identity (ANI) values between the selected *Elizabethkingia* genomes. The scale ranges from 80 to 100% similarity, with values less than 80% depicted in blue and 100% similarity in red. This heatmap illustrates the genetic relatedness and diversity among the different *Elizabethkingia* strains, highlighting the degree of genomic similarity between clinical and environmental isolates. The heatmap was generated using ANI analysis.

The genomes of *E. miricola* Mich-1, Mich-2, Mich-3, and other selected *Elizabethkingia* species were analyzed using ANI and digital DNA–DNA hybridization (dDDH) (Supplementary Table S2). ANI analysis confirmed that the three isolates belonged to the *E. miricola* species, as they shared 96.38% identity with the type strain *E. miricola* DSM 14571 (Figure 3; Supplementary Table S2), exceeding the 95% threshold for species delineation. Furthermore, the ANI values among the three isolates were 100%, indicating they are identical at the genomic level. For comparison, strain CSID_3000516464, also isolated from a Michigan patient in 2016, showed high identity with these strains. Interestingly, isolates from frogs and condensation water exhibited high ANI values with clinical isolates from patients, indicating significant genomic similarity. In contrast, strain BM10, isolated from termites, showed a lower ANI value of 95%, precisely at the species cutoff threshold. ANI values between *E. miricola* and other *Elizabethkingia* species, such as *E. anophelis* and *E. meningoseptica*, were below 86%, confirming their distinction as separate species. The dDDH analysis further supported the ANI findings. The dDDH values for Mich-1, Mich-2, and Mich-3 relative to the type strain *E. miricola* DSM 14571 were 73.5%, exceeding the 70% threshold for species definition. Overall, dDDH results were consistent with the ANI analysis (Supplementary Table S2), reinforcing the classification of these isolates within the *E. miricola* species.

**Figure 4:**
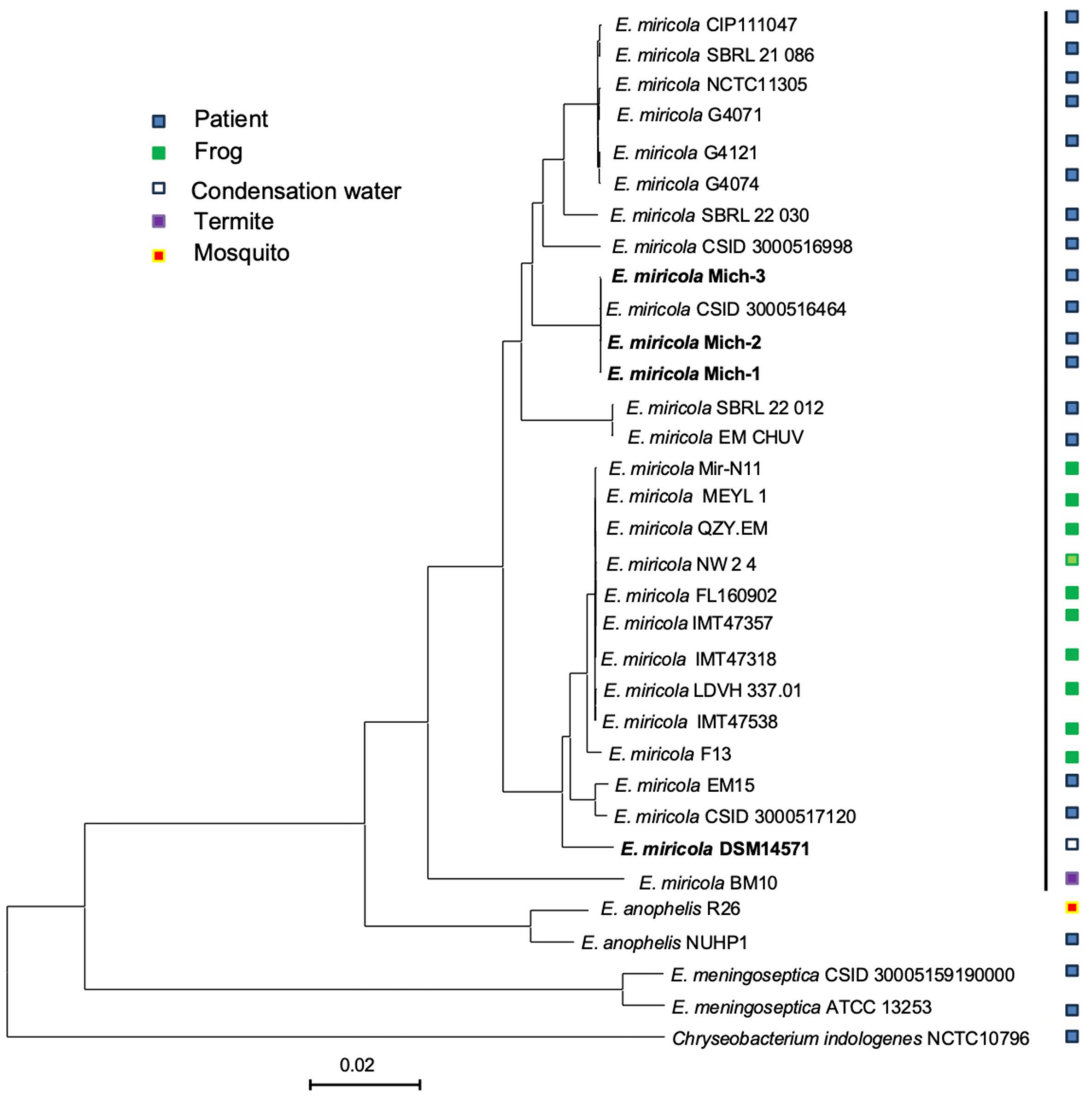
Genome BLAST distance phylogeny tree of selected *Elizabethkingia* genomes. The GBDP tree was constructed based on whole genome sequencing data. The branch lengths represent genetic distances, and the numbers above the branches indicate GBDP pseudo-bootstrap support values (percentages), with values greater than 60% based on 100 replications. The average branch support across the tree is 43.3%. The tree is rooted at the midpoint, providing a phylogenetic overview of the selected *Elizabethkingia* strains and their relatedness.

The four clinical isolates from Michigan patients (*E. miricola* Mich-1, Mich-2, Mich-3, and CSID_3000516464) formed a single clade (Figure 4). Strains SBRL 22-012, SBRL 21-086, and SBRL 22-030, isolated from Ohio between 2011 and 2012, showed distinct phylogenetic placements. Among these, SBRL 21-086 and SBRL 22-030 formed a separate clade with several clinical strains from European countries, while SBRL 22-012 clustered with *E. miricola* EM_CHUV, a strain isolated in Switzerland. Strain *E. miricola* CSID_3000516998 was phylogenetically close to the clade formed by European clinical strains. Interestingly, strain CSID_3000517120, isolated from a Minnesota patient, formed a clade with EM15, a strain from Brazil, within the broader clade of isolates obtained from frogs.

### 3.3 Gene repertoire of *Elizabethkingia miricola*

**Figure 5:**
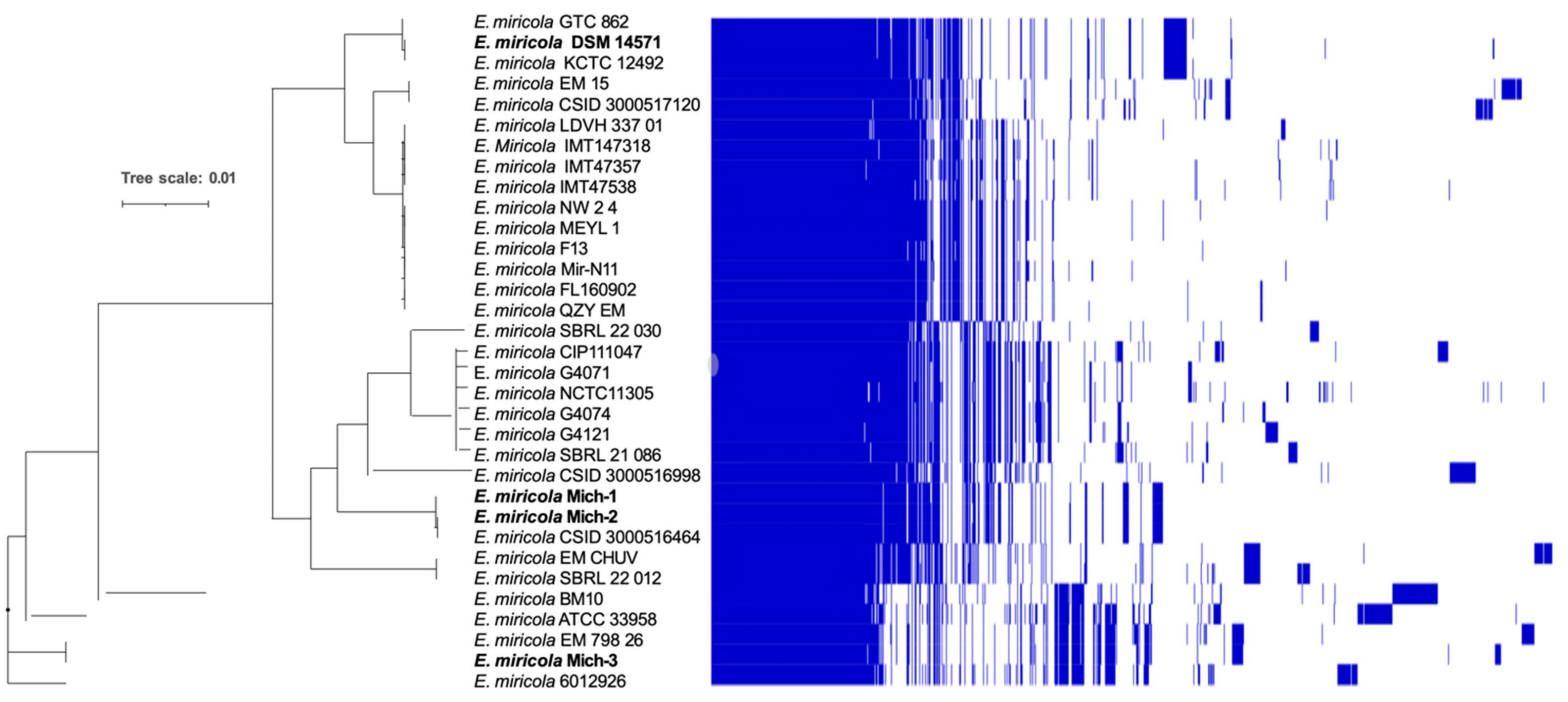
Pangenome analysis of *E. miricola*. Pan-genome presence and absence heatmap combined with a Maximum Likelihood (ML) tree. The genomes of the strains were clustered based on the presence (blue) and absence (white) of genes. This analysis provides a comprehensive view of the core and accessory genes within the *E. miricola* strains. The pangenome visualization was generated using Phandango, offering insights into gene distribution and the genomic diversity of the strains (Page et al., 2015; Hadfield et al., 2018).

The core and pan-genomes of the selected 28 *E. miricola* genomes were analyzed to examine their gene repertoire (Figure 5). The core genome, shared by all the genomes, is typically associated with essential housekeeping functions (Supplementary Table S3). It is further categorized into hard-core genes, present in more than 99% of genomes, and soft-core genes, present in 95 to 99% of genomes. The accessory genome, shared by a subset of genomes, is often linked to pathogenicity or environmental adaptation. This category is further divided into shell genes, present in 15 to 95% of genomes, and cloud genes, present in 0 to 15%. Cloud genes include those unique to individual genomes. The pan-genome of the 28 *E. miricola* isolates comprises a total of 10,944 genes, with 2,201 core genes and 8,743 accessory genes. Within the core genome, 2,025 genes (9%) are hard-core, and 176 are soft-core. The accessory genome is further divided into 2,467 shell genes and 6,100 cloud genes. This genomic organization highlights the balance between conserved elements essential for survival and the diverse accessory components that enable adaptation and pathogenicity.

**Figure 6:**
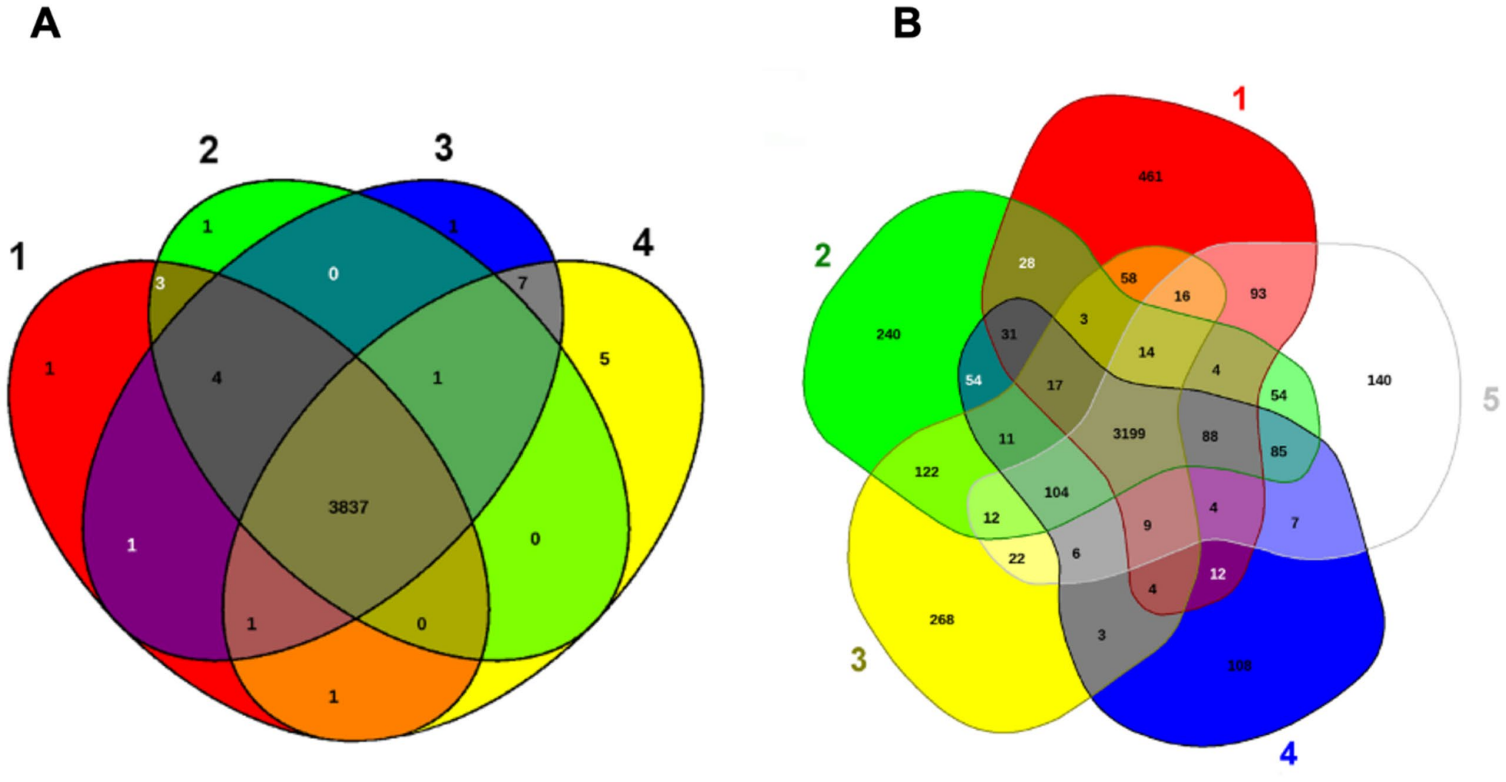
Venn diagram of shared and unique genes of selected *Elizabethkingia* genomes. The unique and shared genome among the selected strains was determined by a dual cutoff of 30% or greater amino acid identity and sequence length coverage of more than 70%. EDGAR was used for Venn diagrams. **(A)** 1: *E. miricola* CSID_3000516464; 2: *E. miricola* Mich-1; 3: *E. miricola* Mich-2; 4: *E. miricola* Mich-3. **(B)** 1: *E. miricola* CSID_3000517120, 2: *E. miricola* SBRL-21-086; 3: *E. miricola* SBRL-21-012 and *E. miricola* SBRL-21-030, 5: *E. miricola* Mich-1.

**Figure 7:**
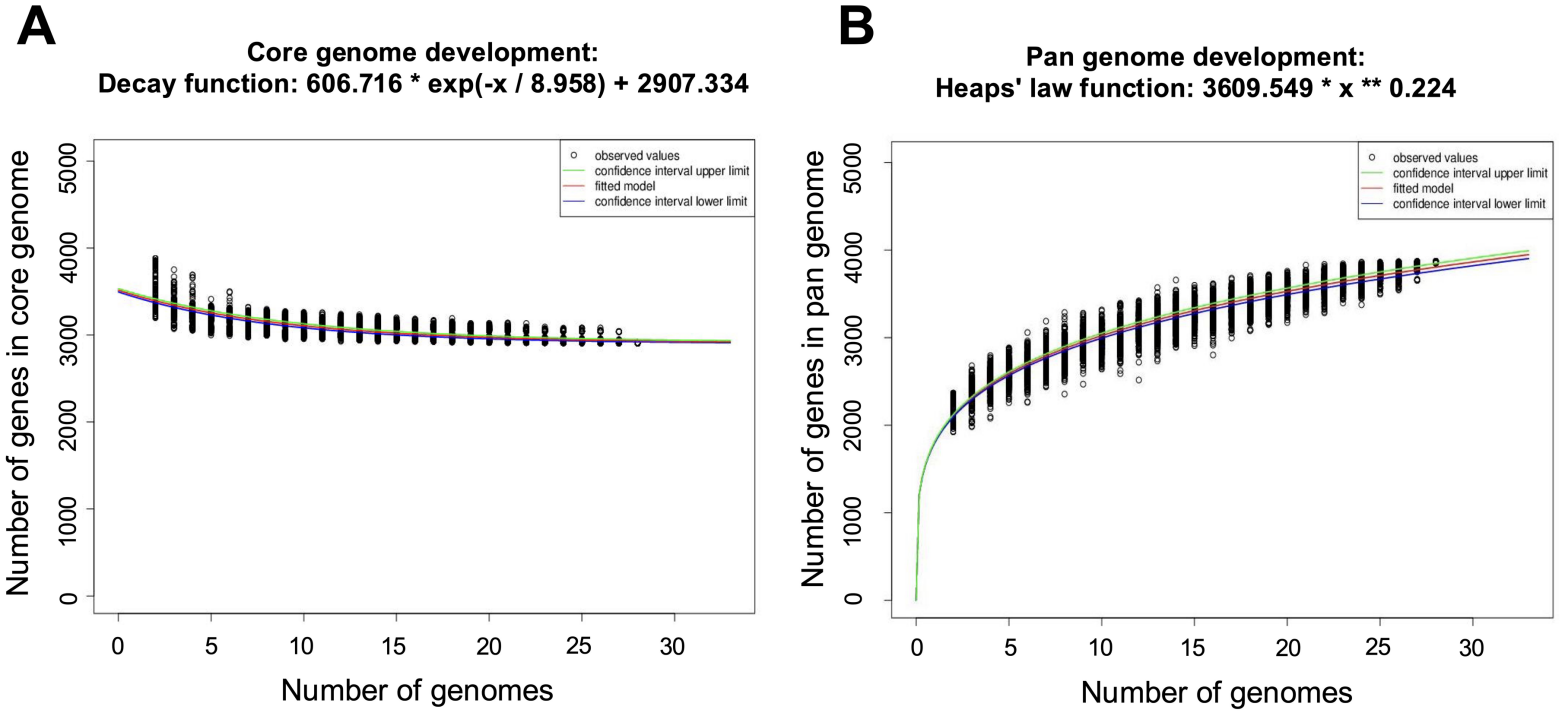
Pan and core genome plots of the 28 selected *Elizabethkingia* genomes. **(A)** Number of core genomes for a given number of genomes sequentially added. **(B)** Number of pan genomes as a function of the number of genomes sequentially added.

*Elizabethkingia miricola* Mich-1, Mich-2, Mich-3, and CSID_3000516464 shared at least 3,837 genes, indicating high genomic similarity among the Michigan isolates (Figure 6A). These strains had only 1 to 5 unique genes, further supporting their close relationship (Figure 6A). However, the genomic content of the Michigan isolates differed significantly from that of isolates from other Midwest regions, such as Ohio and Minnesota. For instance, *E. miricola* Mich-1 shared 3,505, 3,368, 3,558, and 3,427 genes with SBRL-21-030, SBRL-21-012, SBRL-21-086, and CSID_3000517120, respectively, accounting for 91.1, 87.6, 92.5, and 89.1% of its genome. Collectively, Mich-1 and these four strains shared 3,199 genes (83.2%; Figure 6A). Interestingly, Mich-1 shared more genes with frog isolates (3,319 to 3,375 genes) than with some human clinical isolates from the Midwest (Figure 6B). To explore the pan-genome characteristics of the 28 *E. miricola* strains, pan-genome and core-genome curves were generated (Figures 7A,B). The results revealed that the pan-genome expanded significantly as more genomes were included, confirming that *E. miricola* possesses an open pan-genome. Conversely, the core genome size decreased as the number of genomes increased.

### 3.4 Regulatory system proteins

**Figure 8:**
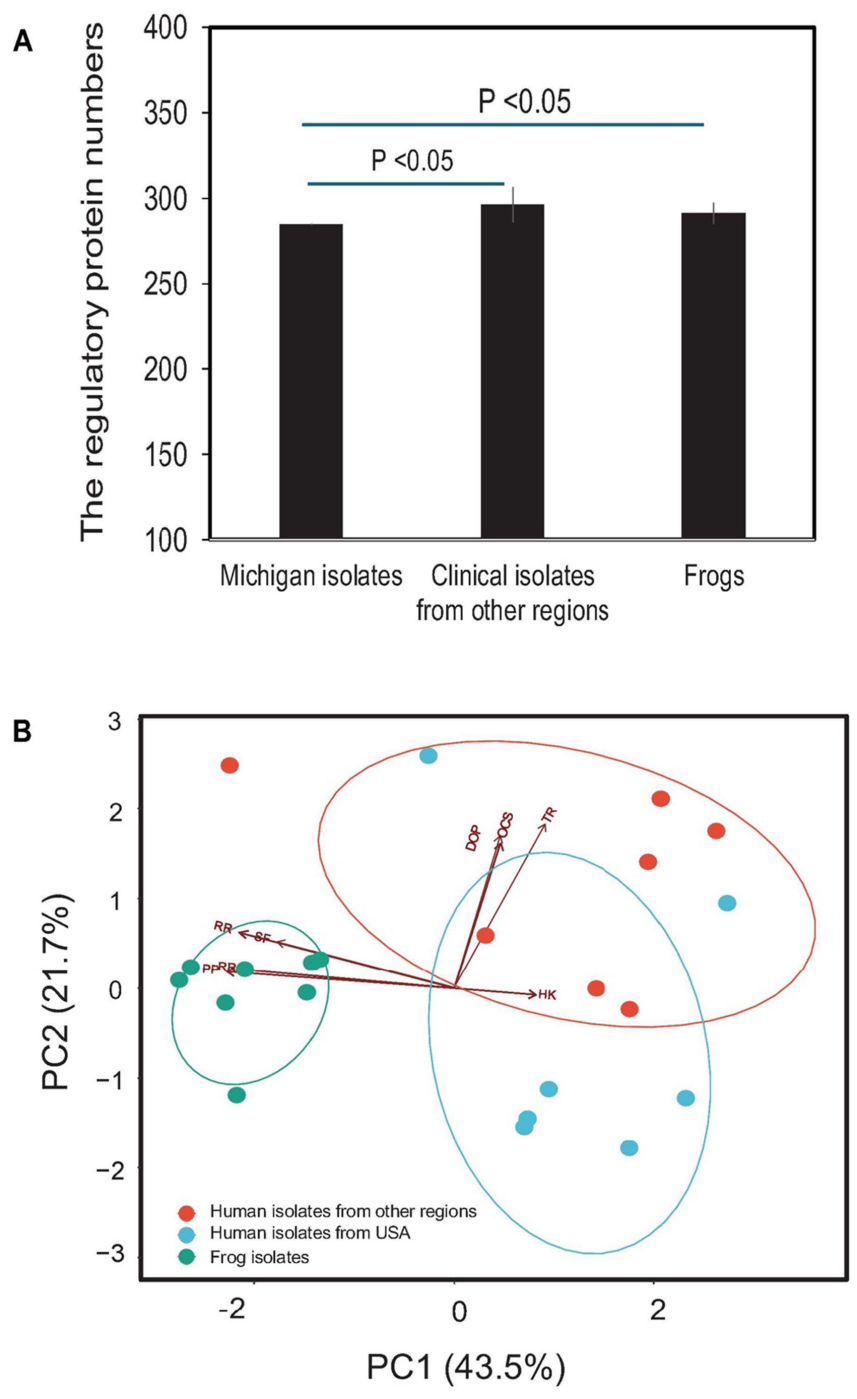
Comparison of predicted regulatory proteins in various *E. miricola* isolates. **(A)** Difference of total regulatory protein numbers between Michigan isolates, other clinical isolates, and frog isolates. **(B)** PCA analysis of various regulatory proteins including response regulators, phosphotransferase proteins, histidine kinases, one-component systems, transcriptional regulators, sigma factors, and other DNA-binding proteins.

The genome of *E. miricola* Mich-1, isolated from Michigan patients, contained genes encoding 63 two-component system proteins, 209 transcription factor proteins, and 10 other DNA-binding proteins, for a total of 282 regulatory proteins (Table 2). Identical counts were observed for Mich-2 and Mich-3. The average total number of regulatory proteins in the Michigan isolates (282) was lower than those from other regions (296) and frog isolates (291) (Table 2). Although the total number of regulatory proteins did not differ significantly between clinical and frog isolates (*p* > 0.05), notable variations were observed in the numbers of response regulators (RR) and sigma factors (SF). These numbers were lower in clinical isolates compared to frog isolates (Table 2; Figure 8A). Principal component analysis (PCA) of regulatory proteins in clinically relevant isolates further revealed a separation between frog-associated isolates and those from human patients (Figure 8B). This distinction highlights potential differences in regulatory adaptations between environmental and clinical *E. miricola* strains.

**Table 2:** Predicted regulatory proteins of selected *Elizabethkingia* genomes.

*Elizabethkingia*	Predicted regulatory proteins							
	Two component systems			Transcription factors				DNA-binding proteins (ODP)
	RR	PP	HK	OCS	RR	TR	SF	
*E. miricola*								
EM-15	36	11	21	31	29	152	21	11
SBRL-21-086	31	7	24	32	27	142	16	12
SBRL-21-012	31	9	22	30	28	132	16	11
SBRL-21-030	30	7	23	32	27	134	16	9
Mir-N11	34	11	24	31	30	139	19	10
NW-2-4	34	11	24	31	30	135	19	10
MEYL_	34	11	24	32	30	134	19	10
ATCC 33958	39	12	23	30	33	154	19	12
FL160902	35	11	25	31	31	131	20	10
IMT47318	34	12	21	32	30	131	20	10
IMT47538	34	11	22	31	30	136	20	10
IMT47357	35	11	22	31	31	133	20	10
QZY. EM	35	11	25	31	31	132	20	10
LDVH-337.01	34	11	22	31	30	127	19	9
CSID_3000516464	32	8	23	29	27	134	18	10
CSID_3000516998	30	7	24	31	27	135	18	8
CSID_3000517120	34	10	22	32	29	146	18	13
G4074	30	7	23	33	26	140	18	13
G4121	31	7	24	33	27	148	19	10
EM_CHUV	34	9	24	32	30	142	16	10
6012926	34	10	22	29	29	145	17	11
EM798-26	33	11	22	26	29	142	19	9
G4071	32	7	23	32	27	142	18	9
NCTC11305	31	8	22	32	25	141	18	9
BM10	29	8	20	24	25	144	18	11
CIP111047	32	7	24	33	27	146	18	12
DSM 14571	32	10	22	31	29	146	19	7
GTC_862	32	10	22	31	29	147	19	7
KCTC 12492	32	10	22	31	29	147	19	8
Mich-1	32	8	23	29	27	135	18	10
Mich-2	32	8	23	29	27	135	18	10
Mich-3	32	8	23	29	27	135	18	10
*E. anophelis*								
NUHP1	28	9	18	29	23	116	18	12
FMS_007	26	8	16	28	22	112	16	11
CSID_30005169	27	8	19	34	24	122	17	10
*E. meningoseptica*								
ATCC	29	10	18	25	25	115	15	6
CSID-300516919000	28	10	19	27	25	123	25	16
EM1	28	10	18	27	25	124	16	7
EM2	28	10	18	26	26	121	16	6
EM3	28	10	18	27	25	124	16	7
G4076	29	10	19	27	25	117	15	6

### 3.5 Resistome analysis

The three Michigan isolates exhibited resistance to at least three distinct categories of antibiotics, including aminoglycosides, nitrofurans, and β-lactams. Notably, even β-lactam/inhibitor combinations, such as ampicillin/sulbactam and piperacillin/tazobactam, failed to enhance drug sensitivity. Among the antibiotics tested, the isolates demonstrated susceptibility only to trimethoprim/sulfamethoxazole (a sulfonamide) and ciprofloxacin (a quinolone), while showing intermediate susceptibility to tetracycline (Table 3). Overall, these isolates displayed multi-drug resistance profiles consistent with those previously reported in *E. meningoseptica* strains isolated from Michigan (Chen et al., 2017, 2019).

**Table 3:** Antibiotic susceptibility tests of Michigan isolates.

Antibiotic class	Tested antibiotics	*E. miricola* Mich-1	*E. miricola* Mich-2	*E. miricola* Mich-3	SIR*
Aminoglycosides					
	Amikacin	≥64	≥64	≥64	R
	Gentamicin	≥16	≥16	≥16	R
β-lactams and β-lactamase inhibitors					
	Meropenem	≥16	≥16	≥16	R
	Cefazolin	≥64	≥64	≥64	R
	Cefotaxime	≥32	≥32	≥32	R
	Tobramycin	≥16	≥16	≥16	R
	Aztreonam	≥64	≥64	≥64	R
	Ampicillin	≥32	≥32	≥32	R
	Ampicillin/Sulbactam	≥32	≥32	≥32	R
	Piperacillin	≥64	≥64	≥64	R
	Ceftriaxone	≥64	≥64	≥64	R
	Piperacillin/Tazobactam	≥128	≥128	≥128	R
Sulfonamide	Trimethoprim/Sulfamethoxazole	40	40	40	S
Quinolone	Ciprofloxacin	1	1	1	S
Tetracycline	Tigecycline	4	4	4	I
Nitrofuran	Nitrofurantoin	128	128	128	R

^*^R, resistant; I, intermediate sensitivity; S, sensitive.

**Figure 9:**
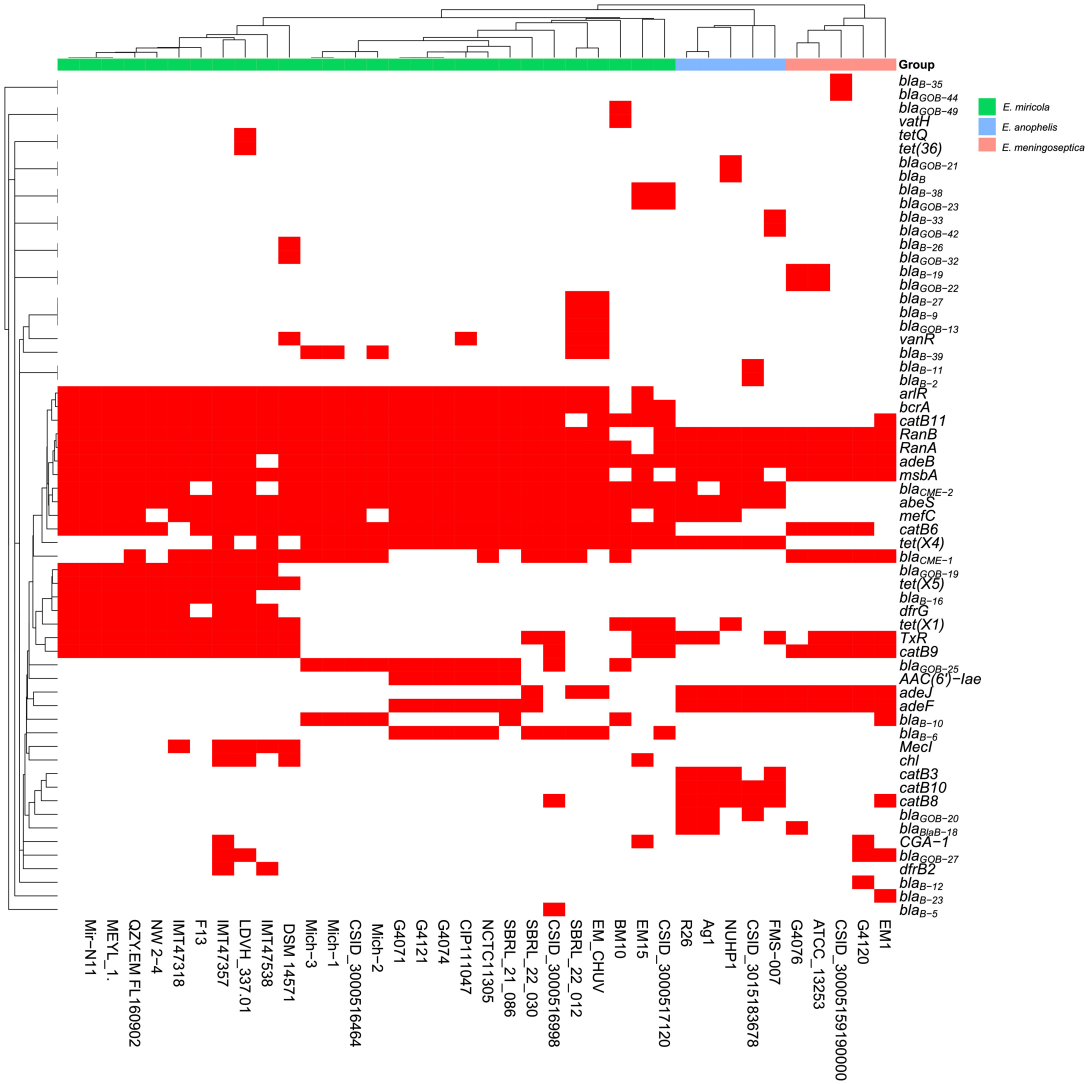
The presence and absence of predicted antibiotic resistance gene based on whole-genome sequence for each isolate. Red squares represented the presence of the related antibiotic resistance genes and white squares represented the absence of the related antibiotic resistance genes.

To correlate the relationship between multidrug resistance phenotypes and their possible genetic determinants, we analyzed the resistance gene profiles in the selected *E. miricola* strains (Figure 9). There was no difference in the distribution of drug-resistance genes among Michigan isolates (Figure 9; Supplementary Table S4). At least five different β-lactamase genes (BlaB-10, BlaB-39, CME-1, CME-2, GOB-25) were found, which may confer their resistance to cephalosporins, penams, and carbapenems. The aminoglycoside resistance gene *aadS* can explain its resistance to aminoglycosides. The presence of *tet(X4)* found in three *E. miricola* genomes and others may partially account for its intermediate sensitivity to tigecycline. Genes encoding several efflux pumps were also discovered, which may confer resistance to nitrofurantoin and other tested drugs in this study (Supplementary Table S4; Table 3). Collectively, certain subtypes of the antimicrobial resistance (AMR) genes were more specifically associated with isolates from either frogs or humans (Supplementary Table S4). For example, GOB-25 (metallo-β-lactamase, subclass B3) was absent in frog isolates. Instead, BlaB-16 was only found in the frog isolates (Supplementary Table S4). However, there were also some shared among these *E. miricola*. Most *E. miricola* strains carried CME-2 (class A β-lactamase) while some carried both of CME-1 and CME-2 (Supplementary Table S4; Figure 9).

### 3.6 Virulence-associated genes of the various *Elizabethkingia miricola* strains

The virulence factors in the selected *Elizabethkingia* species were predicted using the VFDB (Chen et al., 2016). Consistent with previous studies on virulence factors in *Elizabethkingia*, genes involved in capsular polysaccharide biosynthesis, elongation factors, heat shock proteins, phospholipases, catalases, peroxidases, and various other factors were found in nearly all of the selected *E. miricola* genomes (Supplementary Table S5). These genes were reported to participate in stress survival, immune modulation, and environmental adherence (Kukutla et al., 2014; Teo et al., 2014; Breurec et al., 2016; Janda and Lopez, 2017; Perrin et al., 2017; Lin et al., 2019; Zajmi et al., 2022; Andriyanov et al., 2024). For example, *htpB*, *fimH, rmlA* and *EF-Tu* involved in capsule polysaccharide biosynthesis, adherence and invasion were found (Supplementary Table S5). They may facilitate evasion or neutralization of host immune responses and play a pivotal role in biofilm formation and surface colonization (Frees et al., 2004; Zarankiewicz et al., 2012; Hu et al., 2022a; Puah et al., 2022). Iron/heme utilization genes including *iutA* and *hemL* are conserved in most of *E. miricola* (Supplementary Table S5). *E. miricola* also carried many enzymes likely involved in pathogenesis. For example, the *katG* gene, conserved across *Elizabethkingia* species, encodes a catalase-peroxidase heme enzyme known to participate in iron metabolism and stress responses (Ratledge and Dover, 2000; Skaar, 2010; Chen et al., 2020). Urease genes (*ureB* and *ureG*) were found in *E. miricola*, which may play a pivotal role in the pathogenesis of gram-negative bacteria by facilitating survival and colonization in acidic environments (Supplementary Table S5).

## 4 Discussion

*Elizabethkingia miricola* is an emerging pathogen that poses a significant threat to human health (Zajmi et al., 2022; Soler-Iborte et al., 2024; Wu et al., 2024). The infection control of *Elizabethkingia* is challenging due to its intrinsic resistance to many antibiotics, difficult identification, unclear transmission pathways, and ability to persist in healthcare settings (Zajmi et al., 2022; Huang et al., 2024; Wu et al., 2024). Moreover, it can cause various diseases because it has been frequently isolated from the oral cavity, sputum, pulmonary abscesses, urine, CSF, and blood specimens (Choi et al., 2019; Huang et al., 2024). However, little is known about genetic compositions and features of these pathogens (Hu et al., 2020; Wu et al., 2024). To better understand its pathogenic potentials and multi-drug resistance mechanisms, we systematically conducted a comparative genomic analysis between human and frog isolates and its phylogenetic neighbors *E. anophelis* and *E. meningoseptica* (Lin et al., 2019; Puah et al., 2022).

The accurate identification of *Elizabethkingia* to the species level is challenging using morphological observations, routine biochemical tests, and MALDI-TOF MS in the clinical microbiology laboratory (Ransangan et al., 2013; Nicholson et al., 2018; Kadi et al., 2022; Puah et al., 2022). As demonstrated in this study and others, *E. miricola* was misidentified as *E. anophelis* or *E. meningoseptica*. Updates to the latest MALDI-TOF MS libraries are necessary for definitive species identification (McTaggart et al., 2019; Hem et al., 2022; Kadi et al., 2022). 16S rRNA sequence is helpful for molecular identification but it is limited in its taxonomic utility due to the sequence conservation in *Elizabethkingia* (Nicholson et al., 2018; Kadi et al., 2022). For example, Lee et al. reported that the homology of the two 16S rRNA sequences of *E. miricola* BM10 was more than 98% with *E. anophelis* R26, *E. meningoseptica* ATCC 13253, and *E. miricola* GTC 862 (Lee et al., 2019). However, strain *E. miricola* BM10 showed a low ANI value (93.36% identity) with that in three type strains in the genus *Elizabethkingia*, which agrees that it may need to be reclassified to a new genus within *Elizabethkingia*. Therefore, whole genomic sequence analysis, together with ANI or dDDH, may be used to correctly identify *E. miricola* in the future (Eriksen et al., 2017; McTaggart et al., 2019; Hem et al., 2022).

Unlike other *Elizabethkingia* species, *E. miricola* is well known to cause meningitis-like diseases in frogs and cause outbreaks from time to time. Zoonotic transmission from invertebrates to humans is possible but needs more investigation (Ransangan et al., 2013; Zajmi et al., 2022; Huang et al., 2024; Soler-Iborte et al., 2024). Most human isolates formed a different clade from those found in frogs; however, two strains, one from Minnesota and another from Brazil, were phylogenetically close to frog isolates. The genome size, total gene numbers, and average GC contents in isolates from human patients were not significantly different from those isolated from frogs (*p* > 0.05). Moreover, the pan-genomes of *E. miricola* showed that it is evolving through the loss or gain of various genes, which is similar to these observations in *E. anophelis*, *E. meningoseptica*, and other flavobacteria. *E. miricola* lives in diverse environments, including aquatic, terrestrial environments, vertebrates, and invertebrate animals (McTaggart et al., 2019; Hem et al., 2022). It seems that the bacterium adapts to the respective niche environments (Hu et al., 2020; Yang et al., 2021; Soler-Iborte et al., 2024). Thus, it is expected that *E. miricola* has an open pan-genome.

Resistance to β-lactams, tetracycline, and aminoglycosides is particularly concerning, as these drugs are commonly used for treating gram-negative bacterial infections (Hu et al., 2020; Comba et al., 2022; Huang et al., 2024). Comba et al. (2022) reported that 92% (11/12), 50% (6/12), and 83.3% (10/12) of *E. miricola* isolates from the United States were susceptible to piperacillin-tazobactam, ciprofloxacin, and TMP-SMX, respectively (Comba et al., 2022). However, our three isolates were resistant to both piperacillin and piperacillin-tazobactam but remained susceptible to ciprofloxacin and TMP-SMX. In contrast, Wu et al. (2024) found that 100% of 71 *Elizabethkingia* isolates from China were resistant to piperacillin, and 64% were resistant to piperacillin-tazobactam (Wu et al., 2024). These findings suggest that *Elizabethkingia* strains from diverse regions and environments may evolve distinct antibiotic resistance mechanisms (Hu et al., 2022b). However, the clinical significance of these variations remains uncertain due to the lack of standardized interpretive breakpoints for antimicrobial resistance in *Elizabethkingia* spp. (Comba et al., 2022).

A diverse array of drug-resistance genes has been reported in *E. anophelis* and *E. meningoseptica* (Breurec et al., 2016; McTaggart et al., 2019; Comba et al., 2022; Hem et al., 2022; Andriyanov et al., 2024; Wu et al., 2024). However, the profiles of MDR genes have not been comprehensively documented in *E. miricola*. Our results revealed that *E. miricola* harbors two distinct metallo-β-lactamase (MBL) genes (*BlaB* and *GOB*), as well as the CME gene. Remarkably, we found diverse subtypes of the *GOB* and *BlaB* genes in *E. miricola*. Chang et al. (2019) investigated the amino acid sequences of BlaB and GOB and divided them into 22 and 25 different types in *Elizabethkingia*, respectively (Chang et al., 2019). Their phylogenetic analysis showed BlaB and GOB are species-specific proteins. However, the simultaneous presence of both MBL and CME genes may explain its wide resistance to various β-lactams and combination with the β-lactam inhibitors in *Elizabethkingia*. Even novel β-lactamase inhibitors did not significantly enhance the activity of β-lactams (Yasmin et al., 2023). It is possible that those β-lactamase inhibitors may be better on certain β-lactamases while they had little effect on MBLs in *Elizabethkingia* (Kuo et al., 2021). Our discoveries showed that MBLs are intrinsically present in all *E. miricola*, which may confer resistance to Ampicillin/Sulbactam and Piperacillin/Tazobactam (Chang et al., 2019, 2021; Yasmin et al., 2023). Some of them carried several copies of MBL subtypes. Notably, *BlaB*-39 (encoding a class B β-lactamase, subclass B1) was unique to Michigan isolates and *E. miricola* EM_CHUV, suggesting distinct evolutionary pathways. In contrast, *GOB*-25 (subclass B3) was absent in frog isolates, while *BlaB*-16 was exclusively found in frog strains, highlighting unique gene distributions across environments. Two new chromosomal MBL (*blaBlaB*-16 and *blaGOB*-19) variants were found in the genome of *E. miricola* FL160902 (Hu et al., 2020, 2022b). Homologous expression of the two genes in *E. coli* resulted in increased MICs of most β-lactams, including imipenem, meropenem and ampicillin (Hu et al., 2020). *blaGOB*-13 and *blaB*-9 carbapenemase-encoding genes were reported in *E. miricola* EM_CHUV (Kuo et al., 2021). Chen et al. (2024) investigated the individual contributions of *blaB*, *blaGOB* and *blaCME* on MICs of β-lactams in *Elizabethkingia*, showing that the constitutively and highly expressed *blaB* gene significantly increased MICs of carbapenems, decreasing their efficacy *in vivo* (Berlin et al., 2015). Additionally, their studies demonstrated that CME raised MICs for ceftazidime and cefepime (Berlin et al., 2015). Collectively, regardless of allelic combinations, our findings underline the complexity of β-lactam resistance in *E. miricola*. Resistance genes detected in Michigan isolates included *aadS, artR, tex(x4), bcrA*, and *catB6*, which can contribute to the multidrug resistance of *Elizabethkingia* and complicate clinical treatment.

While the mechanisms underlying *Elizabethkingia* pathogenesis remain largely unknown, we found some virulence factors (VFs) involved in adherence, antiphagocytosis and immune evasion commonly conserved in *E. miricola* (Perrin et al., 2017; Hu et al., 2024). For example, the *capD* gene in *E. miricola* strain FL160902 is located in the conserved region of the Wzy-dependent capsule synthesis gene cluster (Hu et al., 2024). Deletion of *capD* results in an impaired capsule structure, notably reducing cell wall thickness. The mutant strain exhibits a significantly lower survival rate in complement-mediated killing assays and an increased capacity to evade macrophage phagocytosis. In frog infection models, the absence of the polysaccharide capsule attenuates virulence. Additionally, *capD* deletion increases bacterial surface hydrophobicity while reducing desiccation resistance and biofilm formation (Hu et al., 2024).

*Elizabethkingia miricola* is well known to cause bloodstream infections and meningitis (Howard et al., 2020; Gao et al., 2021). Therefore, it is important to understand its iron/heme utilization mechanisms in the disease courses (Chen et al., 2015, 2020). *iutA* is part of the *iucABCD-iutA* operon responsible for aerobactin biosynthesis and uptake in *E. miricola* genomes (Chen et al., 2020). Under the iron-limited condition, gene expression of *iutA* was significantly upregulated, indicating its important roles in iron metabolism (Chen et al., 2020). Chen et al. (2020) further demonstrated that deleting the aerobactin biosynthesis gene cluster in *E. anophelis* impairs iron uptake, increases oxidative damage from H_2_ O_2_ , and reduces biofilm formation (Chen et al., 2020). Interestingly, typical alpha or beta hemolysis activity was not observed on sheep blood agar after culturing *E. miricola* strains for 24 h, despite the prediction of a hemolysin gene (*hlyB*). Andriyanov et al. (2024) similarly reported that *E. anophelis* exhibited no hemolysis on 5% bovine blood agar and showed delayed hemolysis on 5% O-human blood agar (Andriyanov et al., 2024). Clear lysis zones appeared only beneath dense colonies with higher biomass (Andriyanov et al., 2024). This suggests that *Elizabethkingia* may employ a slower or more regulated mechanism to break down red blood cells and access iron/heme, possibly preventing excessive host immune activation (Choby and Skaar, 2016; Donegan et al., 2019). If delayed hemolysis enables sustained iron acquisition in vivo, it might help explain the bacterium’s persistence during infections (Choi et al., 2019; Huang et al., 2024). Ureases catalyze the hydrolysis of urea into ammonia and carbon dioxide, which increases the local pH, countering acidic conditions such as those found in the stomach or urinary tract (Konieczna et al., 2012). This process can lead to chronic infections and complications. As in *Helicobacter* (Elbehiry et al., 2025), it is possible that the ammonia produced by urease in *E. miricola* can be toxic to host cells, disrupt epithelial integrity, and elicit an inflammatory response, further aiding in tissue colonization and immune evasion. However, further studies are warranted to elucidate the mechanisms underlying these putative virulence factors.

In conclusion, our study provides the first detailed report on the isolation and characterization of three pathogenic *E. miricola* strains in the United States. All three isolates demonstrated high levels of resistance to most major antimicrobial agent families. Notably, discrepancies between antibiotic susceptibility testing methods underscore the urgent need for standardized interpretative guidelines for *Elizabethkingia* spp., particularly to enhance clinical decision-making. Through comprehensive whole-genome sequencing analysis of *E. miricola* strains isolated from humans, frogs, condensation water, and termites, we identified a diverse repertoire of resistance genes, including *blaB*, *blaGOB*, and *blaCME*, which confer resistance to a broad range of β-lactams. Of particular concern, we identified frog-derived isolates closely related to clinical strains, emphasizing the potential for cross-habitat transmission. Key genes involved in stress regulation, adherence, and immune modulation were conserved across both frog and clinical isolates, further supporting the likelihood of transmission from aquatic environments to clinical settings. This adaptability not only enhances their resistance and virulence but also underscores the significant threats they pose to both human and animal health.

## Data Availability

The datasets presented in this study can be found in online repositories. The names of the repository/repositories and accession number(s) can be found at: https://www.ncbi.nlm.nih.gov/genbank/, JBEUGN000000000, JBEUGO000000000, and JBEUGP000000000.

## Footnotes

^1^ https://usegalaxy.org/
