# Supplementary material for: Molecular characterization, comparative genome analysis and resistance determinants of three clinical Elizabethkingia miricola strains isolated from Michigan
Source: Front Microbiol. 2025 Jul 9;16:1582121. doi: 10.3389/fmicb.2025.1582121 (PMC12283610; doi:10.3389/fmicb.2025.1582121)
Supplement: Supplementary file 1 [file Data_Sheet_1.zip › Table 1.docx]

=

|  | 1 | 2 | 3 | 4 | 5 | 6 | 7 | 8 | 9 | 10 | 11 | 12 |
| --- | --- | --- | --- | --- | --- | --- | --- | --- | --- | --- | --- | --- |
| A | **Negative Control**  No Growth | **Dextrin**  +++ | **D-Maltose**  + | **D-Trehalose**  + | **D-Cellobiose**  - | **Gentiobiose**  ++ | **Sucrose**  - | **D-Turanose**  - | **Stachyose**  - | **Positive Control**  +++++ | **pH 6**  +++++ | **pH 5**  - |
| B | **D-Rafinose**  - | **α -D-Lactose**  + | **D-Melibiose**  + | **β-Methyl-D-Glucoside**  _ | **D-Salicin**  - | **N-Acetyl-D-Glucosamine**  ++ | **N-Acetyl-β-D-Mannosamine**  - | **N-Acetyl-D-Galactosamine**  - | **N-Acetyl-Neuraminic Acid**  - | **1% NaCl**  +++ | **4% NaCl**  _ | **8% NaCl**  - |
| C | **D-Glucose**  ++ | **D-Mannose**  ++ | **D-Fructose**  ++ | **D-Galactose**  + | **3-Methyl Glucose**  + | **D-Fucose**  + | **L-Fucose**  +++ | **L-Rhamnose**  + | **Inosine**  + | **1% Sodium Lactate**  +++++ | **Fusidic Acid**  - | **D-Serine**  ++++ |
| D | **D-Sorbitol**  - | **D-Mannitol**  ++ | **D-Arabitol**  + | **Myo-Inositol**  - | **Glycerol**  + | **D-Glucose-6-PO_4_**  + | **D-Fructose-6-PO_4_**  ++ | **D-Aspartic Acid**  - | **D-Serine**  - | **Troleandomycin**  ++ | **Rifamycin SV**  ++++ | **Minocycline**  - |
| E | **Gelatin**  +++ | **Glycyl-L-Proline**  ++ | **L-Alanine**  + | **L-Arginine**  + | **L-Aspartic Acid**  ++ | **L-Glutamic Acid**  + | **L-Histidine**  + | **L-Pyroglutamic Acid**  + | **L-Serine**  ++ | **Lincomycin**  + | **Guanidine HCl**  +++ | **Niaproof 4**  - |
| F | **Pectin**  + | **D-Galacturonic Acid**  ++ | **L-Galactonic Acid Lactone**  + | **D-Gluconic Acid**  - | **D-Glucuronic Acid**  +++ | **Glucuronamide**  + | **Mucic Acid**  + | **Quinic Acid**  + | **D-Saccharic Acid**  + | **Vancomycin**  + | **Tetrazolium Violet**  ++++++ | **Tetrazolium Blue**  ++++++ |
| G | **p-Hydroxy-Phenylacetic Acid**  - | **Methyl Pyruvate**  - | **D-Lactic Acid Methyl Ester**  - | **L-Lactic Acid**  - | **Citric Acid**  + | **α-Keto-Glutaric Acid**  - | **D-Malic Acid**  - | **L-Malic Acid**  - | **N-Bromo-Succinic Acid**  - | **Nalidixic Acid**  +++ | **Lithium Chloride**  +++ | **Potassium Tellurite**  +++ |
| H | **Tween 40**  **++** | **γ -Amino-Butyric Acid**  - | **α-Hydroxy-Butyric Acid**  - | **β-Hydroxy-D,L-Butyric Acid**  - | **α -Keto-Butyric Acid**  - | **Acetoacetic Acid**  + | **Propionic Acid**  - | **Acetic Acid**  + | **Formic Acid**  - | **Aztreonam**  ++++ | **Sodium Butyrate**  ++ | **Sodium Bromate**  - |

**Table S1 Biolog tests of *E. miricola***
