# Supplementary material for: Molecular characterization, comparative genome analysis and resistance determinants of three clinical Elizabethkingia miricola strains isolated from Michigan
Source: Front Microbiol. 2025 Jul 9;16:1582121. doi: 10.3389/fmicb.2025.1582121 (PMC12283610; doi:10.3389/fmicb.2025.1582121)
Supplement: Supplementary file 1 [file Data_Sheet_1.zip › Presentation 1 (51).pptx]

## Slide 1
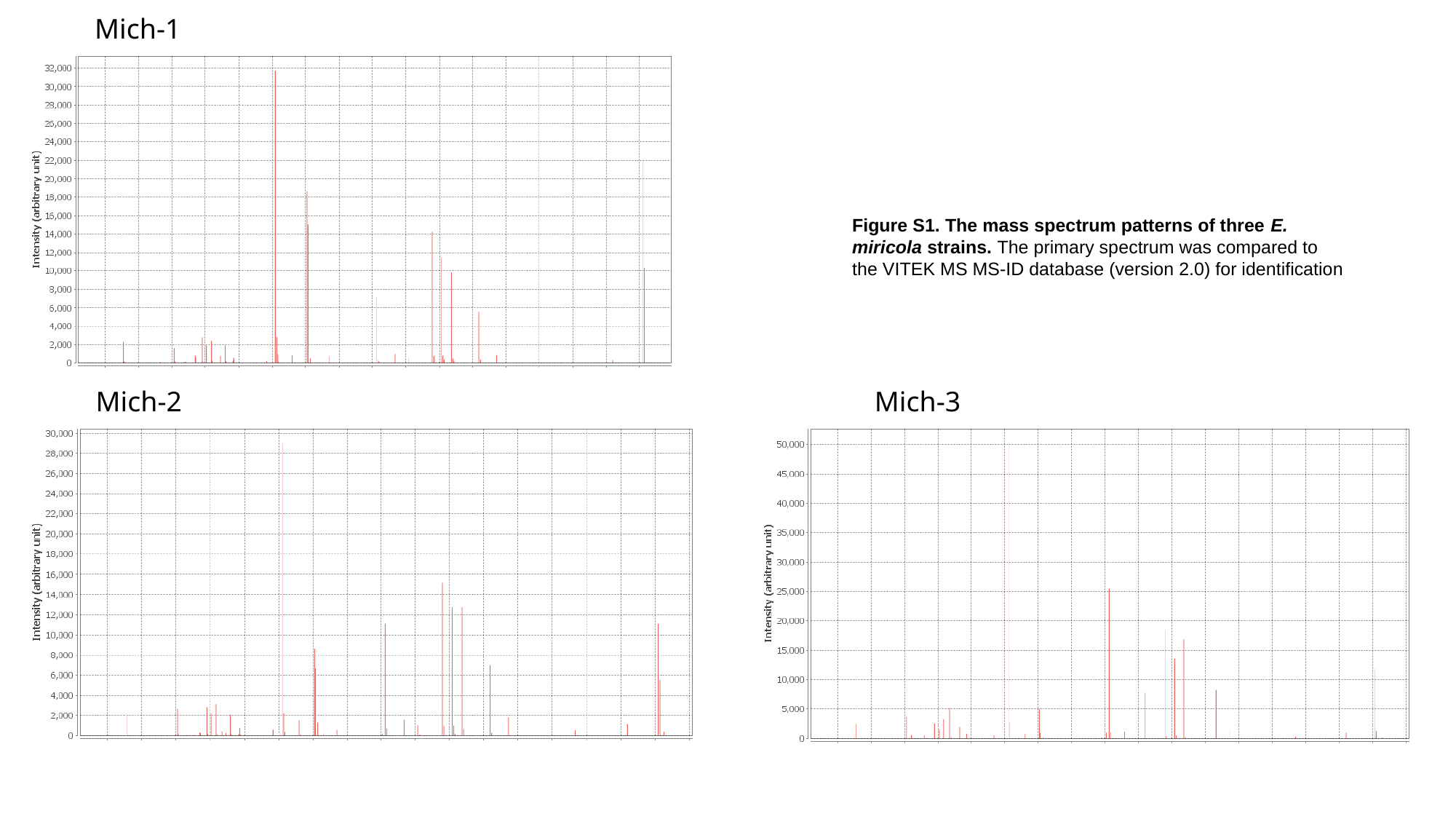

Mich-1
Figure S1. The mass spectrum patterns of three E. miricola strains. The primary spectrum was compared to the VITEK MS MS-ID database (version 2.0) for identification
Mich-2
Mich-3
